# Novel Retrograde Tibial Intramedullary Nailing for Distal Tibial Fractures

**DOI:** 10.3389/fsurg.2022.899483

**Published:** 2022-05-10

**Authors:** Bin Peng, Teng Wan, Wenfu Tan, Weiming Guo, Min He

**Affiliations:** ^1^ The Second Affiliated Hospital, Department of Orthopaedic Surgery, Hengyang Medical College, University of South China, Hengyang, Hunan, China; ^2^ Hengyang Medical College, University of South China, Hengyang, Hunan, China; ^3^ Sports medicine department, Huazhong University of Science and Technology Union Shenzhen Hospital; the 6th Affiliated Hospital of Shenzhen University Health Science CenterShenzhen, China

**Keywords:** distal tibia, fracture, retrograde tibial intramedullary nail, surgery, retrospective study

## Abstract

**Purpose:**

Postoperative distal tibial fractures are often associated with complications such as difficulties in fracture healing and surgical incision infection. The purpose of this study is to evaluate the fracture healing time and functional recovery after a retrograde tibial intramedullary nail treatment for distal tibial fractures.

**Methods:**

We retrospectively studied 9 cases of patients with distal tibial fractures treated with retrograde intramedullary nailing (IMN). Fracture healing time was based on monthly postoperative x-ray imaging results, and functional outcomes were defined according to the American Orthopaedic Foot and Ankle Society (AOFAS) ankle-hindfoot score.

**Results:**

Among the 9 patients with distal tibial fractures from July 2020 to April 2021, the mean age was 51.8 ± 13.8 years. The classification of distal tibial fractures in the 9 patients according to OTA (Orthopaedic Trauma Association) includes 6 extra-articular fractures (3 of type A1, 2 of type A2, and 1 of type A3) and 3 intra-articular fractures (1 of type C1 and 2 of type C2). Among them, there were 5 closed fracture cases and 4 open fracture cases (according to Gustilo classification: 2 of type I, 2 of type II). We treated the fractures surgically with new retrograde tibial intramedullary nailing. The mean follow-up time for this group was 7.9 months (5–12 months). According to monthly postoperative radiographs performed to monitor fracture healing, the mean healing time was 3.3 months (3–4 months). Final postoperative function according to the AOFAS ankle-hindfoot score shows 6 excellent cases, 3 good cases. No serious complications such as postoperative infection, bone and internal fixation exposure, osteofascial compartment syndrome, or vascular nerve injury occurred.

**Conclusion:**

The new retrograde tibial intramedullary nail (RTN) has some unique advantages, and its treatment of distal tibial fractures can achieve good efficacy, but further studies are still needed to verify it.

## Introduction

Distal tibial fractures are a relatively common type of injury in clinical practice, accounting for approximately 10% of all tibial fractures (1, 2). Due to the proximity of the distal tibia to the ankle joint, fracture repositioning, fixation difficulties, poor local soft tissue coverage and blood supply are currently a challenging clinical problem (3). Common surgical treatment options include reduction and internal fixation, and reduction and internal fixation with anterograde tibial intramedullary nails. However, both have certain shortcomings. Compared with plates, the anterograde tibial nail (ATN) reduces the impact on local soft tissue and periosteal blood flow. However, due to the gradual widening of the distal tibial medullary cavity, poor repositioning and instability of fixation are likely to appear (4–6). In recent years, Kuhn et al. designed a novel retrograde tibial intramedullary nail, which provides a new option for minimally invasive treatment of distal tibial fractures (7). Until now, there is no report about the clinical application of new RTN. In this article, we monitored fracture healing time and recorded postoperative ankle functional outcomes after distal tibial fracture surgery and observed the occurrence of postoperative complications.

## Methods

Inclusion criteria: age ≥18 years and distal tibial epiphysis closed; diagnosis of distal tibial fracture, i.e. fracture within 11 cm of the articular surface of the distal tibia; closed fracture or open fracture of Gustilo type I or II; according to OTA (Orthopaedic Trauma Association) fracture classification: 43-A1/A2/A3, 43-C1/C2. Exclusion criteria: combined with ipsilateral tibial proximal or middle tibial stem fracture; pathological fracture or old fracture; ankle deformity; local skin and soft tissue defect and infection; combined vascular and nerve injury requiring repair; patients with poor basic conditions, combined with multiple medical disorders and unable to tolerate surgery. In the retrospective study, from July 2020 to April 2021, we analyzed a total of 9 patients. The RTN is provided by Double Medical Technology Inc (China), which is called metal interlocking intramedullary nail with the registration number of 20153131195. The nail was 8 mm in diameter and 140 mm in length.

**Figure 1:**
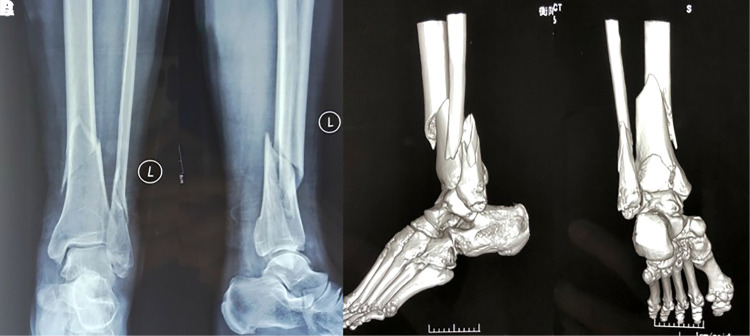
Typical case: female, 54age, fall injury, OTA fracture staging type 42-C1. (**A**) Preoperative X-ray orthopantomograph of distal tibial fracture; (**B**) Preoperative X-ray lateral radiograph of distal tibial fracture; (**C,D**) 3D CT of ankle showing posterior ankle fracture.

**Figure 2:**
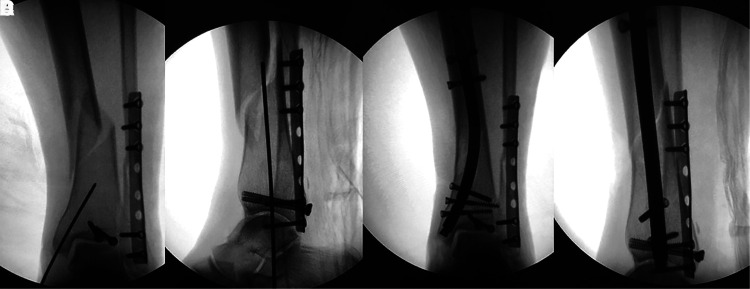
(**A,B**) firstly, the posterior lateral ankle approach was taken, and plates and tension screws were applied to fix the fibula and posterior ankle fractures (RTN guide pins at the medial ankle site), and the orthogonal and lateral films were positioned under intraoperative fluoroscopy, respectively; (**C,D**) a medial minimally invasive incision was used for RTN placement to fix the tibial metaphysis fracture, and the orthogonal and lateral films were taken, respectively.

**Figure 3:**
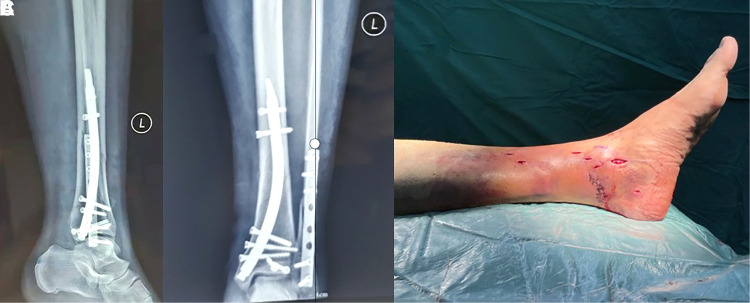
(**A**) X-ray orthopantomogram at one month after1 closed reduction RTN internal fixation of distal tibial fracture; (**B**) X-ray lateral radiograph at one month after1 closed reduction RTN internal fixation of distal tibial fracture; (**C**) surgical incision of the affected limb after closed reduction RTN internal fixation of distal tibial fracture.

All surgeries were performed by the same orthopedic surgeons. If fibula fractures affect the stability of the ankle (within 8 cm above the malleolar fossa), open reduction and plate fixation of the fibula are performed first via the lateral approach. In patients with intra-articular fractures, the distal tibial articular fragments were fixed with lag screw, then the treatment of metaphyseal fractures with RTN were followed.The 2 cm long incision started at the tip of the medial malleolus with sharp dissection and separation. Under the protective sleeve device, a guide wire was inserted and confirmed by C-arm fluoroscopy. The insertion point was at the midpoint of the medial malleolus in both anteroposterior (AP) and lateral radiographs. Moreover, the insertion direction was parallel to the medial cortex in AP fluoroscopy, which located at the anatomical axis of the distal tibia in lateral fluoroscopy. Along the the guide wire, a cannulated awl was used to create a hole and path until reaching the medullary canal. Then, the guide wire and awl were moved and RTN was assembled on the aiming device. With low force and small twisting movements, the nail was introduced into the the medullary canal until the end was flush with the cortex of medial malleolus. C-arm fluoroscopy served to confirm the reduction and correct nail position. If satisfactory, the proximal and distal locking screws were placed through the the trocar combination, and an end cap was introduced at the nail end finally. If the fracture had significant angulation or lateral displacement, the nail was returned and the fracture was corrected by some reduction techniques. For example, blocking screws, percutaneous clamps at the fracture site, and open clamp application with minimal incision were all viable. The typical case is shown in Figures 1–3.

Postoperative anticoagulation was performed for 24 h, and functional ankle exercises were started 2–3 days after surgery. Regular follow-up and monthly postoperative x-ray imaging was performed to monitor fracture healing. Further rehabilitation and weight bearing guided by review results. At the final follow-up, if the angle of the distal tibial coronal plane was greater than 5°, the ankle was considered to have an inversion or valgus deformity. The clinical outcome was also evaluated using the AOFAS ankle-hindfoot score

## Results

A retrospective study of 9 patients with distal tibial fractures with new retrograde tibial intramedullary nailing for closed reduction and internal fixation was performed (Table 1). The patients, 4 male and 5 female, are followed up for mean 7.9 months (5–12 months). The age range of the included patients was 18–69 years with a mean of 51.8 years. There are 3 cases with left-sided fracture and 6 cases with right-sided fracture. 5 cases are caused by traffic injury and 4 cases are caused by falls. The fractures were classified according to OTA classification, with 6 cases of extra-articular fractures (A1, A2, A3) and 3 cases of intra-articular fractures (C1, C2). The length of the tibial stem fracture line from the articular surface was 7.9–10.8 cm, with an average of 9.1 cm. There were 5 closed fractures and 4 open fractures (according to Gustilo typing: 2 cases of type I and 2 cases of type II). Nine patients had a combined fibula fracture, one patient had a combined thoracic trauma and one patient had a combined craniocerebral trauma. The time between injury and RTN surgery ranged from 5 to 16 days, with a mean of 11.4 days. Plates were used for internal fixation in 4 cases with fibula fracture. The mean healing time was 3.3 months (3–4 months) according to monthly postoperative X-ray imaging. All achieved clinical healing of the fracture and returned to daily life and work successfully. The imaging evaluation showed no angulation of the distal tibial coronal plane greater than 5°, and no inversion deformity occurred. Ankle-hindfoot score according to AOFAS indicated 6 excellent cases, 3 good cases. No patients had serious complications, including infection, bone and internal fixation exposure, osteofascial compartment syndrome, or vascular nerve injury.

**Table 1:** Patient data for closed reduction of distal tibial fracture with new retrograde tibial intramedullary nail internal fixation.

Medical Record Number	Gender	Age (years)	Injury types	Left / Right	AO/OTA Fracture Types	Length of fracture line from joint surface (cm)	Open fracture/Gustilo typing	Combined injuries	Fracture healing Time (month)	The angulation of the distal tibial coronal plane (°)	AOFAS Rating
1	Female	57	Traffic Injuries	Right	43-A1	7.9	Not applicable	Cranial Trauma	3	3	Excellent
2	Male	18	Fall injury	Left	43-A1	8.7	Not applicable	None	3	5	Excellent
3	Female	69	Traffic Injuries	Right	43-C2	10.2	Type I	None	4	2	Good
4	Male	54	Fall injury	Right	43-C2	10.8	Type II	None	3	4	Excellent
5	Male	66	Traffic Injuries	Right	43-A2	9.4	Type II	None	4	2	Good
6	Male	45	Traffic Injuries	Left	43-A2	8.3	Not applicable	Chest Trauma	4	0	Excellent
7	Female	52	Traffic Injuries	Right	43-A3	9.2	Type I	None	3	3	Excellent
8	Female	54	Fall injury	Left	43-C1	9.6	Not applicable	None	3	0	Excellent
9	Female	51	Fall injury	Right	42-A1	8.2	Not applicable	None	3	2	Good

## Discussion

Distal tibial fractures are more common in clinical practice and have specific anatomic features (1,2). The cross-sectional morphology of the tibia changes from trigonous to quadrangular in the middle and lower third of the tibia, and the medullary cavity becomes progressively wider. In addition, the soft tissue around the distal tibia is less and very heterogeneous, and there is no muscle attachment on the anterior medial side. And its local extramedullary blood supply is relatively insufficient, while the trophoblastic vessels in the medullary cavity are relatively homogeneous. Therefore, the local resistance to infection and bone healing of the distal tibia is poor, and infection, delayed fracture healing and non-healing are likely to occur. High-energy injuries are often accompanied by severe soft tissue injuries or open fractures, which makes the management of distal tibial fractures more difficult. In our series, most were high-energy injury cases, and some of them had hyperpigmentation of the lower extremity due to chronic diseases such as varicose veins, which undoubtedly made the treatment more difficult.

Currently, plates and intramedullary nails are the two main forms of internal fixation, and many studies have shown no significant difference in the overall efficacy of the two treatment modalities (3, 8, 9). However, plate fixation is more demanding on soft tissue conditions. Although percutaneous minimally invasive plate technique can reduce soft tissue stripping, we still need to be alert to this serious problem, especially in patients with poor local soft tissue conditions. In recent years, with the continuous development of the ATN technique, its indications have been extended to distal tibial fractures with satisfactory results. However, due to the gradual widening of the distal tibial medullary cavity, the special anatomical structure prevents the intramedullary nail from fitting tightly into the medullary cavity, which makes the fracture prone to poor repositioning and lack of stability, resulting in malunion and delayed healing (10). Therefore, ATN treatment of distal tibial fractures often requires a combination of reduction techniques such as blocking screws (11, 12), but this undoubtedly increases the difficulty and technical requirements of the procedur. In addition, it also has some adverse effects on the knee joint (13).

In 2014, Kuhn et al. reported a new type of RTN with a distally curved banana-shaped main nail and multiple interlocking nails at both ends to form a multi-axial fixation (7). For the indications of RTN, it has been noted that it can be used for extra-articular fractures of the distal tibia and some intra-articular fractures (OTA typing: 43-A1/A2/A3, 43-C1/C2 types) (14). In our group, there were 6 cases of extra-articular fractures (3 cases of A1 type, 2 cases of A2 type, 1case of A3 type) and intra-articular fractures accounted for 3 cases (1 case of C1 type, 2 cases of C2 type), and all patients obtained good results. For intra-articular fractures, we first applied tension screws to fix the intra-articular bone block, and then applied RTN to treat distal tibial epiphysis fractures. In addition, RTN may be the only intramedullary nailing option for patients combined with internal fixation of ipsilateral tibial plateau fractures or total knee replacement.

Through literature review and preliminary clinical application, we believe that the new RTN has some unique advantages. First, In terms of biomechanics, the RTN has better resistance to rotation and similar axial stability compared with the ATN (15). Second, compared with the distal medial tibial plate, the RTN has both better rotation and axial stability (16). It is well known that good biomechanical stability is one of the necessary conditions for fracture healing, and all patients in this group successfully achieved the fracture healing, which also reflects the good stability of the RTN. Third, because the intramedullary nail has a distal curved banana shape, it is more compatible with the morphology of the distal tibia. In the initial clinical application, we believe that the fracture repositioning and fixation is simpler and more convenient by using RTN. In all cases, only 1 patient required an adjunctive repositioning technique such as blocking nail, and postoperative radiographic measurements showed no occurrence of distal tibial valgus deformity. Forth, RTN used the medial ankle as the entry point, thus avoiding anterior knee pain, and postoperative results showed that it also had less impact on the ankle joint. However, RTN may have a risk of distant ankle pain and susceptibility to fracture, which needs to be verified with a larger sample size and long-term follow-up. Fifth, due to the shorter RTN, its locking nail aiming device has a high accuracy, and all the locking nails in this group of patients could be accurately placed one first time, which relatively reduced the operation time and radiation exposure. Also, the shorter intramedullary nail did not pass through the isthmus of the tibial trunk and had relatively less impact on the bone marrow cavity. This may have reduced the risk of fat embolism, especially in patients with combined lung injury or multiple injuries. However, we currently have a small number of cases and further studies are needed for confirmation.

## Conclusion

In conclusion, the novel RTN for distal tibial fractures can achieve satisfactory efficacy and has some unique advantages. However, we need to increase the number of cases and follow-up time and conduct further prospective randomized controlled studies to provide a more solid basis for the wide application of RTN.

## Data Availability

The original contributions presented in the study are included in the article/Supplementary Material, further inquiries can be directed to the corresponding author/s.
